# A novel dual mechanism-of-action bispecific PD-1-IL-2v armed by a “βγ-only” interleukin-2 variant

**DOI:** 10.3389/fimmu.2024.1369376

**Published:** 2024-04-04

**Authors:** Yongji Jiang, Chuyuan Chen, Yuan Liu, Rong Wang, Chuan Feng, Lili Cai, Shuang Chang, Lei Zhao

**Affiliations:** ^1^ Division of AAV Discovery, Department of Gene Therapy, Cure Genetics Co., LTD, Suzhou, China; ^2^ Division of Research & Development, Department of Cell Therapy, Cure Genetics Co., LTD, Suzhou, China

**Keywords:** interleukin-2, cytokine, bispecific antibody, T cell activation, cancer immune therapy, IL-2 variant

## Abstract

**Introduction:**

Interleukin-2 (IL-2) is one of the first cytokines to be discovered as an immune agonist for cancer immunotherapy. Biased IL-2 variants had been discovered to eliminate Treg activation or enhance the tumor specific T cell cytotoxicity. However, all the biased IL-2 variants pose the risk to overstimulate immune response at a low-dose range. Here, we introduce a novel dual-MOA bispecific PD-1-IL-2v molecule with great anti-tumor efficacy in a high dosed manner.

**Methods:**

The novel IL-2 variant was designed by structural truncation and shuffling. The single armed bispecific PD-1-IL-2v molecule and IL-2v were studied by immune cell activations in vitro and in vivo and anti-tumor efficacy in mouse model.

**Results and discussion:**

The IL-2 variant in this bispecific antibody only binds to IL-2Rβγ complex in a fast-on/off manner without α, β or γ single receptor binding. This IL-2v mildly activates T and NK cells without over stimulation, meanwhile it diminishes Treg activation compared to the wild type IL-2. This unique bispecific molecule with “βγ-only” IL-2v can not only “in-cis” stimulate and expand CD8 T and NK cells moderately without Treg activation, but also block the PD-1/L1 interaction at a similar dose range with monoclonal antibody.

## Introduction

Interleukin-2 (IL-2) is a pluripotent cytokine produced primarily by activated CD4+ T cells, which plays a crucial role in orchestrating the proliferation, survival, and function of immune effector cells (Teff), regulatory T cells (Treg) and natural killer (NK) cells to maintain immune homeostasis (1–3). The recombinant human IL-2 was first approved by the U.S. Food and Drug Administration (FDA) in the 1990s for the treatment of metastatic renal cell carcinoma and metastatic melanoma. However, the clinical use of IL-2 has been limited by its therapeutic window, which has been restricted by both low-dose induced immunosuppression and high-dose induced toxicity. This “two-way” immune regulative property depends on its multiple binding patterns with three individual subunits IL-2Rα (CD25), IL-2Rβ (CD122), γ (common gamma chain, γc, or CD132) (4). Low-dosed IL-2 prefers to bind a heterotrimeric complex of IL-2Rαβγ expressed on Treg cells with a high affinity (Kd=10^-11^ M), therefore it down-regulates the inflammation by activation of Treg. High-dosed IL-2 can bind a heterodimeric complex of IL-2Rβγ subunits expressed on CD8 T and NK cells with an intermediate affinity (Kd=10^-9^ M), it enhances the pro-inflammation by activation of effective T cells (Teff) and NK cells (5). Moreover, high-dosed IL-2 also binds the monomeric IL-2Rα or IL-2Rβ subunit, which characteristically expressed on the endothelial cells or NK cells respectively, with relatively low affinity (Kd=10^-8^ M). Therefore, the over activation of endothelial cells or NK cells by high-dosed IL-2 are believed as the main reasons of VLS (6, 7) or over stimulation of immune system (8, 9).

To reduce the risk of immunosuppression and VLS from the activation of Treg and endothelial cells, respectively, many “no-α” (βγ biased) IL-2 variants have been discovered (10). The strategies of “no-α” IL-2 design are 1) by the shield of CD25 binding surface of IL-2 such as NKTR-214 (11), THOR-707 (12) and ALK4230 (13) etc.; 2) by the mutation of CD25 binding domain of IL-2 such as RO6895882 (14, 15), MDNA11 based on the “superkine” (16) etc.; 3) by *de novo* designed molecule without CD25 binding like NL-201 (17). Although the no-α IL-2 variants significantly reduce the risk of VLS and Treg activation, the tolerable clinical doses of those variants are less than 10 μg/kg (normalized to the content of IL-2) compared with 37 μg/kg of wtIL-2. The toxicities of no-α IL-2 variants may be associated with over stimulation of immune system (9).

Recently, “no-α” IL-2 variants had been used in the “*in-cis*” T cell activation by a fusion design with a T cell targeting antibody. For instance, RO7284755, a PD-1-IL-2v bispecific antibody developed by Roche (18), yielded a better effect on stimulation of stem-like CD8 T cells. This “*in-cis*” agonist is currently in clinical trial (NCT04303858) in combination with a PD-L1 antibody. The dosage of this “*in-cis*” agonist is about 1/10 of PD-L1 mAb in pre-clinical mouse model, indicating that the moiety of PD-1 antibody in this bispecific molecule only contributes to T cell targeting but not for PD-1/L1 blocking, so that it required PD-L1 combination to complement the PD-1/L1 blockage (19).

To develop an ideal “*in-cis*” PD-1-IL-2v with functions from both cytokine and checkpoint inhibitor, the dosage should increase to mAb level. Thus, the IL-2v moiety should not be recruited by any of its receptors (known as IL-2Rα and IL-2Rβ) but retain IL-2Rβγ binding affinity to moderately activate CD8 T and NK cells. Here, we report a “βγ-only” IL-2 variant followed by the procedure of 1) truncation of IL-2Rα binding surface; 2) shuffle the 4 α-helix to obtain the re-constructed molecules and 3) circular permutated the shuffled molecules to generate a small library. Our selected IL-2 variant has no α or β single receptor binding affinity that remove the Treg activation and NK over-stimulation, but remains similar βγ complex binding affinity compared with wtIL-2. Moreover, this variant binds to IL-2Rβγ in a “fast-on/off” manner to avoid deep-activation of Teff, which helps to maintain T cell stemness and reduce exhaustion after cell expansion. By fusion with PD-1 antibody, this bispecific PD-1-IL-2v demonstrates a robust anti-tumor efficacy at a high-dose level (mAb level) in comparison with the same dose of PD-1 antibody, indicating its potential dual-MOAs (mechanism of actions) as a safe and efficacious “*in-cis*” IL-2 agonist with PD-1/L1 blocking function.

## Materials and methods

### Plasmid construction, protein expression and purification

All expression plasmids were constructed using the pET30a vector. Wild type IL-2 was mutated to include de-alanine-1, C58S and C125S. After the steps of truncation, shuffling, circular permutation and point mutations, 24 IL-2 polypeptides were generated (**Supplementary Table S1**). The wildtype IL-2 and all 24 variant plasmids were transformed to *E. coli* BL21 (DE3) and stimulated by 0.5 mM IPTG to express the proteins. The bacteria were then harvested and re-suspended in the lysis buffer (50 mM Tris-HCl, 5 mM EDTA, 1 mM PMSF, pH 8.0), which was collected after the high-pressure homogenizer (ATS Engineering Inc., AH1500). After centrifugation (12000 rpm, 30 min), the precipitates were collected (except 041, 042 and 043), washed with washing buffer (50 mM Tris-HCl, 5 mM EDTA, 300 mM NaCl, pH 8.0; 2 h, 3 times). For 041, 042 and 043, after cell lysis and centrifugation, the supernatants were collected and treated with saturated ammonium sulphate solution. The precipitates were then washed using the lysis buffer for 2 times. After washing, the inclusion bodies were de-natured in the solubilization buffer (50 mM Tris-HCl, 5 mM EDTA, 1 mM PMSF, 9 M Urea, pH 8.0) by mild shaking for 2 h. Urea concentration and pH of solubilization buffer were adjusted for certain variants. After centrifugation at 13000 rpm for 30 min, the supernatants were dropped in the 9 times volume of refolding buffer (50 mM Tris-HCl, 1 mM EDTA, 10% (w/v) sucrose, 1 mM PMSF, pH 8.0) and stood overnight at 4 °C. The solutions were then centrifuged at 13000 rpm at 4 °C for 30 min. The supernatants were filtered with 0.22 μm diameter filter and stored at 4 °C. For 041, 042 and 043, the supernatants were treated by the XS/Q chromatography purification, the eluted fragments were collected. The proteins were further concentrated by centrifugation at 5000 rpm at 4 °C for 15 min as needed. The concentrations of the proteins were determined by BCA, Bradford kit, and/or SDS-PAGE. The thermo-stability of the purified IL-2 variants at 37 °C were monitored by OD350 for 48 hours. The final IL-2 variants which used in biological assays was also identified by ACQuity H-class Plus Xevo G2-XS Qtof for the molecular weight.

The bispecific PD-1-IL-2v fusion protein was expressed in CHO S cell, the knob-into-hole plasmids 0.5 ug/mL (VH1:VH2:VL=2:2:3) were added into 40 uL/mL buffer (OptiPRO SFM, Gibco) followed by mixing the transfection buffer (ExpiFectamine™ CHO Transfection Kit, Gibco with 36.8 uL/mL OptiPRO SFM) for less than 5 min. All buffer were transferred to the medium (EmCD CHO-S 203 medium) in 37°C and 8%CO_2_ for 7 days. The Feed (240 uL/mL) and Enhancer (6 uL/mL) were added to expression medium after 16-22 h of transfection to feed the CHO S cell. After protein expression, the supernatant of medium was collected followed by filtration (0.45 um). The product was then purified by Protein A column and SP FF with standard protocol.

### *In vitro* activity assays of IL-2 variants

The *in vitro* activities of the purified IL-2 variants were first confirmed by STAT5 activation assay using NK92 (expressing high-affinity receptor α/β/γ) and HH cell lines (expressing intermediate-affinity receptor β/γ). NK-92 cells were plated into 12-well plates at 500,000 cells/well in IL-2-deprived media (MyeloCult^®^ H5100). 12 hours later, different doses of wtIL-2 or IL-2 variants were added into well-plate. Similarly, HH cells were also plated into 12-well plates at 500,000 cells/well in full media (RPMI 1640 medium with 10% FBS). Different doses of wtIL-2, IL-2 variants, or bispecific PD-1-IL-2v were also added. The samples were incubated at 37 °C, 5% CO_2_ for 30 minutes. The cells were collected by centrifugation at 1500 rpm for 5 min and lysed using Laemmli Buffer. The bioactivities of the IL-2 variants were indicated by STAT5 phosphorylation as measured by western blot and quantified by the pSTAT5: total STAT5 ratio.

Human PBMCs and mouse splenocytes were separately treated with the wild-type IL-2 and the IL-2 variants, respectively, and STAT5 phosphorylation was measured by western blot. The activation profiles of the IL-2 molecules were indicated by % pSTAT5, which was calculated by the relative intensity of pSTAT5 to STAT5, normalized with that of the sample treated wild-type IL-2 at high concentration (25 nM).

### The binding affinity measurement of surface plasmon resonance and ELISA

The IL-2 receptor proteins were immobilized on a Biacore™ sensor chip CM5 according to manufacturer’s protocol. Wild-type IL-2 and IL-2 variant 012 at different concentrations were passed over the surface of the chip in a stepwise model. Different concentrations of wild-type IL-2 and IL-2 variant 012 were flowed over the bound ligand on the surface of the chip for 120 seconds, after which a blank solution was passed over the surface to allow the IL-2R to dissociate. The resulting sensorgrams were analyzed with the native instrument software to calculate the binding affinities.

The binding affinity of human PD-1 and bispecific PD-1-IL-2v has been measured by ELISA. The PD-1 has been coating on the plate (1000 ng/mL, 100uL/well) overnight at 4°C. The well was then washed by PBS with 0.5% tween-20 for 3 times followed by adding the PD-1-IL-2v at 100 uL/well. After washing 3 times by the same wash buffer, the HRP-goat anti human IgG (1:10000) was added to well for 1 h followed by the detection of OD450-OD570 nm.

### Expansion of hPBMC by using wtIL-2 and IL-2v

To confirm the function of IL-2v in expansion of hPBMC, a cell proliferation assay was performed. PBMCs from healthy donors were cultured in the presence of CTS™ Dynabeads™ CD3/CD28 (Gibco 40203D) and either 012 or 001 for 4 days, followed by 8 more days culture with the same IL-2 molecule (IL-2 variant or wild type) but without anti-CD3/CD28 Dynabeads. The cell type of CD4+ T cell, CD8+ T cell, Treg, NK cell had been identified in FACS stained by particular antibody. The stemness profile of each cell type after expansion were monitored by stemness markers such as CCR7 and CD62L after 10 days expansion while the exhaustion markers such as PD-1, LAG-3 and TIM-3 were monitored on day 12 by flow cytometry.

### RNAseq for the expanded CD8+ T cell and NK cell by wtIL-2 and IL-2v

The CD8 T cell and NK cell were used to test gene transcription by RNAseq. The cells were expanded by following the protocol of Miltenyi Biotec CD8 MicroBeads human (order No. 130-045-201). wtIL-2 and IL-2v were used in the expansion process at 50 nM. The expanded cells were sent to Azenta Life Science for RNAseq test, the results were analyzed by FPKM (Fragments Per Kilo bases per Million reads), the differentiation of gene pathway annotation were based on KEGG.

### *In vivo* toxicity and immune cell activation of wtIL-2 and IL-2v

The toxicity of the IL-2 variants was determined in comparison with the wild-type IL-2. Specifically, 12 groups of normal female Balb/c mice (n=6 per group) received IP injections of (1) vehicle [phosphate-buffered saline (PBS)], (2) 0.1 mg/kg, 0.3 mg/kg, 1 mg/kg, 3 mg/kg or 10mg/kg rhIL-2 (Beijing Four Rings Bio-Pharmaceutical, Recombinant Human Interleukin-2 for Injection, Lot. S10970015) in PBS or (3) 0.1 mg/kg, 0.3 mg/kg, 1 mg/kg, 3 mg/kg, 10mg/kg or 30mg/kg 012 in PBS, respectively, once daily for 5 days. Mouse body weight was measured on Day 3 and Day 6. Additionally, pulmonary edema resulting from capillary leak in mice was assessed by measuring the increase in wet pulmonary weight. On the morning of the 6th day, mice were euthanized. The lung tissue samples were dehydrated by incubation at 65°C for 72 hours. The resulting dry lung weight was recorded and the weight from pulmonary fluid (wet lung weight) was calculated. Furthermore, the IL-5 level had also been monitored to identify the toxicity by kit. The spleens of the mice treated as described above were harvested, and lymphocyte populations were analyzed by flow cytometry to identify the expansion of immune cells.

### *In vivo* antitumor efficacy

Efficacy studies of IL-2v single agent and combination treatment of IL-2v and PD-1 were performed using female C57BL/6 mice (18–20 g) obtained from Charles River Laboratories (Beijing, China), n = 6/group. For the MC38 model, female C57BL/6 mice (18–20 g) were implanted in the right flank with 5x10^5^ cells in 0.1 mL of phosphate-buffered saline (PBS). Treatment was initiated when the mean tumor size reached 80 – 100mm^3^. In IL-2v single agent study, animals were administered vehicle, 012, or 001 by an intraperitoneal injection. The wtIL-2 doses were 1 mg/kg and 3 mg/kg, while 012 doses were 10 mg/kg, 30 mg/kg, and 60 mg/kg, qdx5 then rest for 2 days and for another cycle. In the combination treatment with IL-2v and PD-1 study, anti-mouse PD-1 dose was 10 mg/kg (qw) and IL-2v doses were 3 mg/kg, 10 mg/kg and 30 mg/kg (tiw) for 4 cycles. The *in vivo* efficacy study of bispecific PD-1-IL-2v was using humanized PD-1 mice with the dose of anti-human PD-1 or PD-1-IL-2v in 30 mg/kg (qw) in 4 cycles. After tumor cell inoculation, the animals were checked daily for morbidity, mortality, effects of tumor growth, and effects of treatment on normal behaviors and appearance such as mobility, food and water consumption, eye/hair matting, and any other abnormal effects. Body weight gain/loss was measured three times weekly. Tumor volumes were measured three times weekly in two dimensions using a caliper, and the volume was expressed in mm^3^ using the formula: V = 0.5 a x b2 where a and b are the long and short diameters of the tumor, respectively. All animal studies were conducted under accreditation by the Association for Assessment and Accreditation of Laboratory Animal Care (AAALAC). Animals were housed in individualized ventilated cages under controlled conditions of temperature, humidity, and light and fed a diet of C060 irradiation sterilized dry granule food. All mice were adapted in the facility for at least 7 days. Euthanasia criteria consisted of either body weight drop of > 20% for 72 hours, hypoactivity, hypothermia, respiratory distress, tumor volume reaching 3500 mm^3^ or termination of the study on day 21.

### Immunohistochemistry of tumor tissue

The immunohistochemistry (IHC) of tumor tissue in the *in vivo* efficacy experiment was provided after euthanasia of mice and harvest the solid tumor. The IHC sample preparation and the staining assay were then followed by the standard protocol (20, 21).

## Results

### Generation, validation and selection of “βγ-only” IL-2v

**Figure 1:**
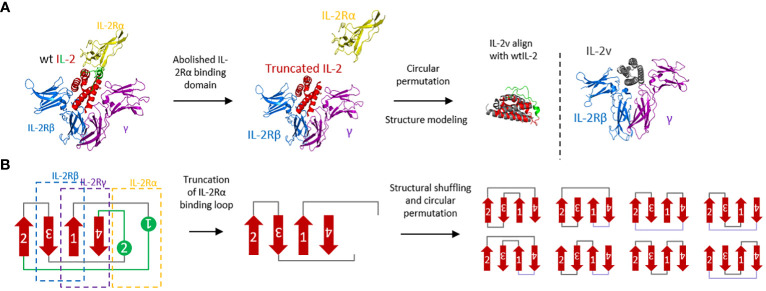
Molecular design and protein engineering of wtIL-2. **(A)** Structural complex with IL-2Rα (yellow), IL-2Rβ (blue), common γ chain (purple) and wtIL-2 (red and green) (PDB:2B5I). The green part of wtIL-2 indicated the binding loop with IL-2Rα. The structural modeling of IL-2v is using AlphaFold2. **(B)** Topological presentation of engineering of wtIL-2.

To generate the “βγ-only” IL-2 variant, the binding affinity with single IL-2 receptor α and β was removed by 1) truncation of the loop structure in IL-2 which interacting with IL-2Rα and 2) generating the molecular tension by structure shuffling and circular permutation, respectively (**Figure 1**). Additional mutations were then introduced to increase the expression yield and protein stability, including de-alanine-1, C58S, and C125S. Moreover, other basic mutations, including P65D, F117K and/or F124K were also introduced to further stabilize the helixes and decrease the hydrophobicity of surface (the number of each amino acids was based on wild-type IL-2 sequence **Supplementary Table S1**). After protein expression and purification (**Supplementary Figure S1**), wild-type IL-2 and IL-2 variants were then validated by expression yield, thermo-stability and *in vivo* activity (**Supplementary Figures S2**-**S6**, **Supplementary Table S2**).

**Figure 2:**
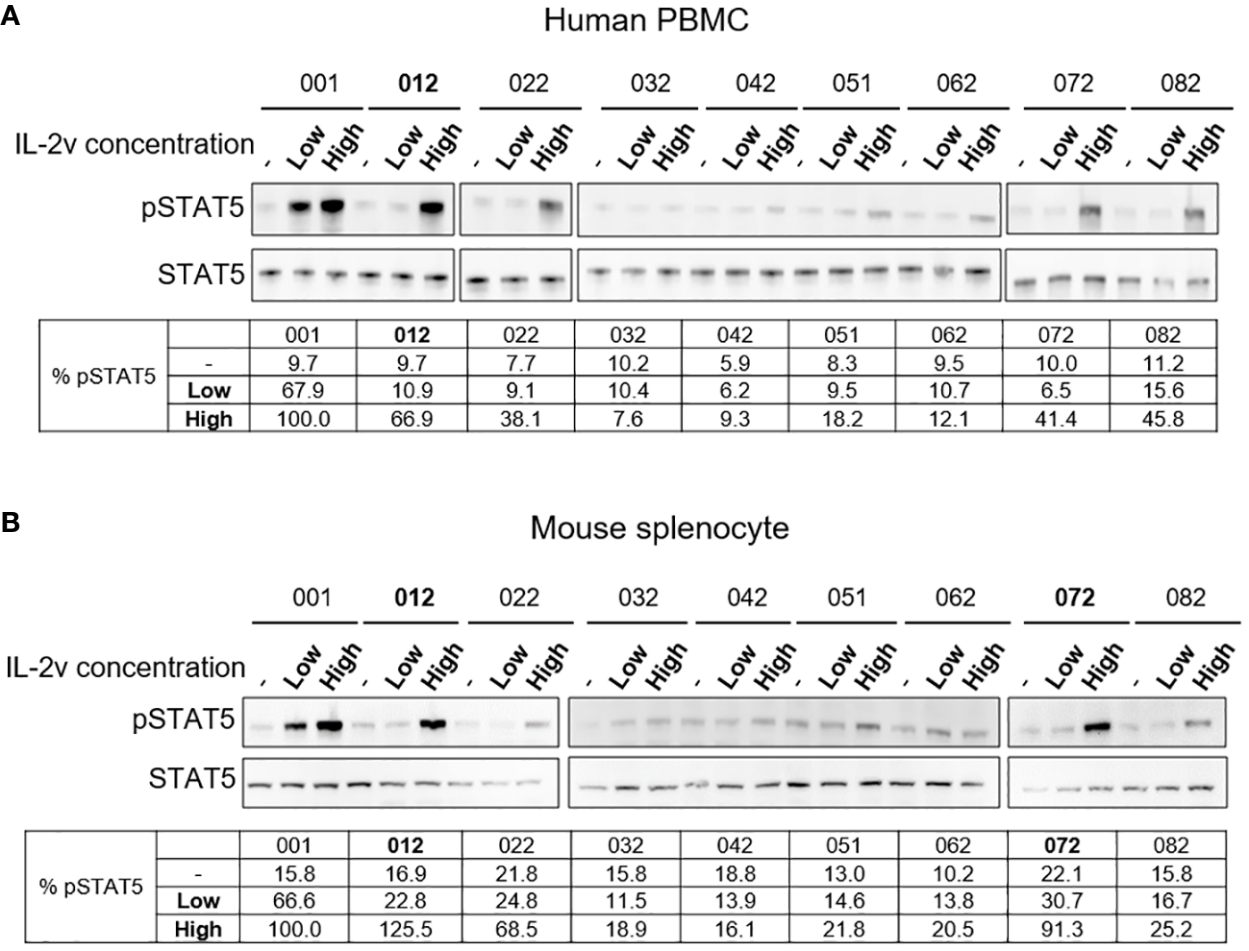
Western blot results of wtIL-2 and variants for pSTAT5 activation. Results for **(A)** human PBMC and **(B)** mouse splenocyte assays for low (2.5 nM) and high (25 nM) concentration treatment by wtIL-2 or IL-2 variants.

As showed in **Supplementary Table S2**, IL-2 variants 012, 022, 032, 042, 051, 062, 072 and 082 were selected for next step validation using human PBMC and mouse splenocyte assays to confirm the binding property of βγ but “no-α”. As shown in **Figure 2**, treatment by wild-type IL-2 at low concentration (2.5 nM) resulted in about 65% activation of pSTAT5 signaling in both healthy donor human PBMCs and naive Balb/c mouse splenocytes. By contrast, samples treated by IL-2 variants at low concentration (2.5 nM) showed very little activation of pSTAT5 signaling (about 10% or less), indicating a loss of preference to high-affinity receptors, or reduced affinity to trimeric receptor IL-2Rαβγ. Nonetheless, comparable activation was observed in samples treated with certain IL-2 variants at high concentration (25 nM) as compared to the wild-type treatment. As shown in **Figure 2A**, treatment by IL-2 variant 012 resulted in comparable activation as treatment by wild-type IL-2 in PBMC. As shown in **Figure 2B**, treatment by variants 012 and 072 resulted in comparable activation as treatment by wild-type IL-2 in mouse splenocytes. These results confirmed that IL-2 variant 012 retained its function to bind and activate the intermediate-affinity receptor IL-2Rβγ, but lost the preferential binding to the high-affinity receptor IL-2Rαβγ. Therefore, 012 was chosen as “βγ-only” IL-2 variant candidate in the following investigations. The right molecular weight of 012 was also confirmed by QTof (**Supplementary Figure S7**).

**Figure 3:**
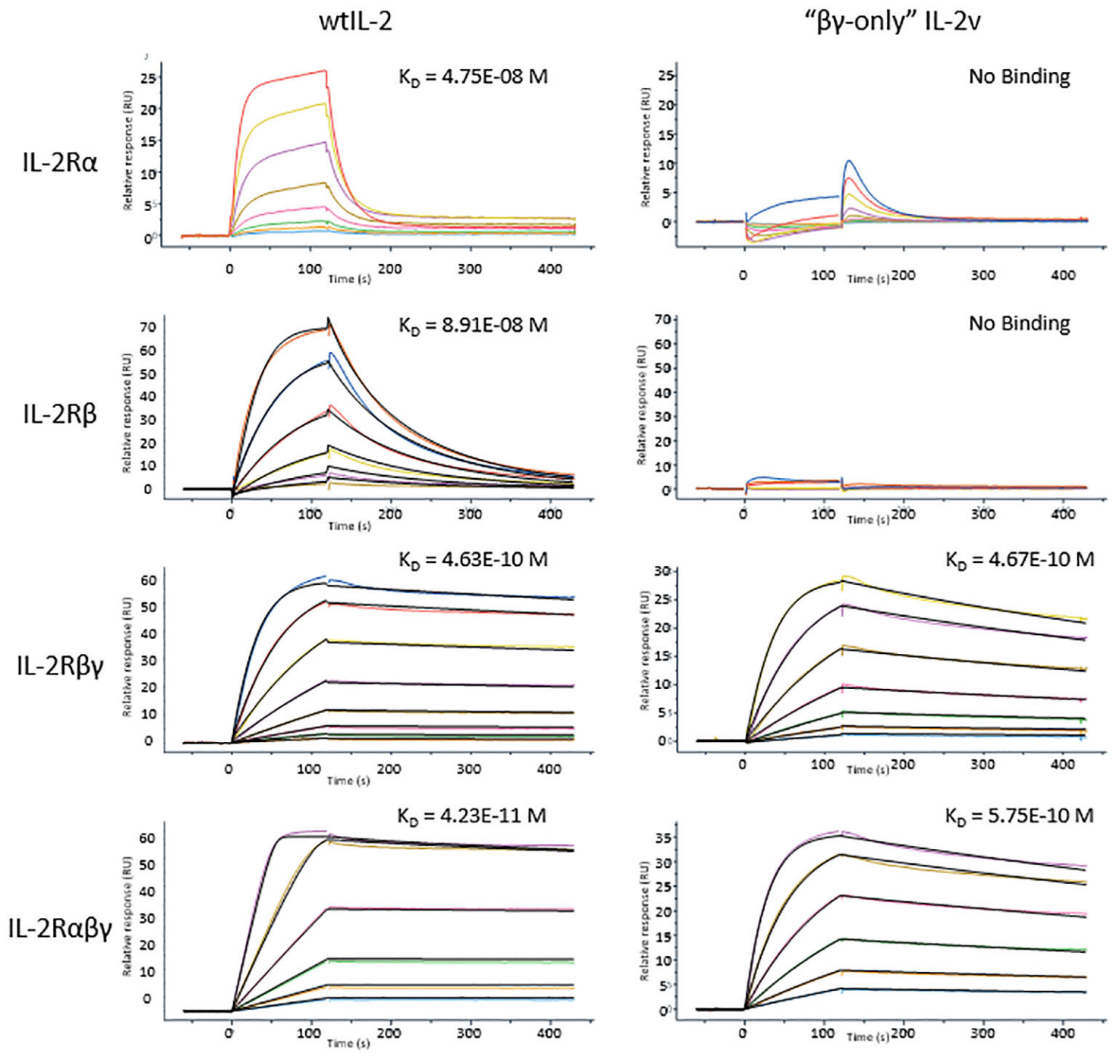
SPR results of wtIL-2 or IL-2v binds to each IL-2 receptors or their complexes. The K_D_s are highlighted on each curve of SPR test.

To confirm the “βγ-only” binding profile, wild-type IL-2 and IL-2 variant 012 was tested in surface plasmon resonance (SPR) with the binding affinity to IL-2Rα, IL-2Rβ, common γ receptor, IL-2Rβγ and IL-2Rαβγ. As shown in **Figure 3**; **Supplementary Table S3**, the binding affinities of the IL-2 variant 012 to single IL-2 receptors (IL-2Rα, IL-2Rβ, and common γ receptor) as well as IL-2Rαβγ were reduced approximately 10-fold compared to those of the wild-type IL-2, whereas IL-2 variant 012 bounds to the IL-2Rβγ receptor at an equally high affinity as the wild-type. Interestingly, this “βγ-only” binding pattern represented around 3 times faster binding kinetics (k_on_ and k_off_) than wtIL-2 which binds to IL-2Rβγ complex (**Supplementary Table S3**) which is a “fast-on, fast-off” feature indicating a unique binding mechanism of IL-2 variant 012.

### “βγ-only” IL-2v indicated the low Treg response and moderate CD8 T and NK activation *in vitro*

**Figure 4:**
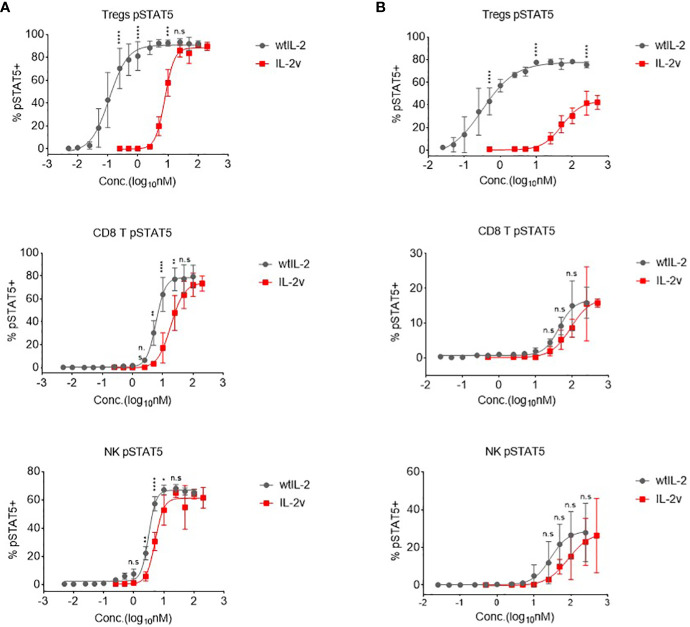
*In vitro* pSTAT5 signaling activated by wtIL-2 and IL-2v. **(A)** pSTAT5 signal in human PBMCs. **(B)** pSTAT5 signal in mouse splenocytes. The Tregs, CD8 T and NK cells were monitored (3 replicates/group).

To determine the selective activation profiles of IL-2v in primary cells, pSTAT5 was measured in different cell populations in the healthy donor human PBMCs and the naive Balb/c mouse splenocytes, respectively. As shown in **Figure 4**, the potencies of IL-2v and wtIL-2 to activate STAT5 signaling differed around 70-fold in the CD4+FOXP3+ Treg population of human PBMCs. IL-2v induced pSTAT5 in Tregs with an EC50 of 7.909 nM, while wtIL-2 induced pSTAT5 with an EC50 of 0.1122 nM (**Figure 4A**; **Supplementary Table S4**). By contrast, similar potencies were observed for IL-2v and wtIL-2 treatment in non-activated CD8 T and NK cells, which expressed the IL-2Rβγ complex. Similarly, in mouse splenocytes, IL-2v showed dramatically decreased potencies in Tregs, as compared to wtIL-2 (**Figure 4B**; **Supplementary Table S4**), but had similar EC50s to that of wtIL-2 in CD8 T and NK cells. As such, IL-2v was much less potent in inducing STAT5 activation in Tregs but maintains similar potency in CD8+ T and NK cells as compared to wtIL-2.

### “βγ-only” IL-2v moderately expanded CD8 T and NK cells, but not Tregs, *in vitro/vivo*, which exhibits an excellent *in vivo* safety profile

**Figure 5:**
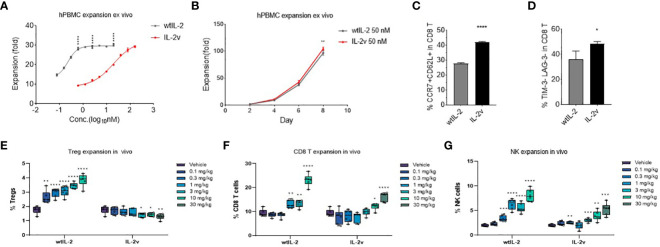
Cell expansion by wtIL-2 or IL-2v *ex vivo/in vivo*. The total cell expansion were monitored in **(A)** different concentration or in **(B)** different expansion time by treatment of wtIL-2 or IL-2v *ex vivo* (3 replicates/group). The stemness marker CCR7 and CD62L as well as the exhaustion marker TIM-3 and LAG-3 have been monitored in **(C, D)**, respectively (3 replicates/group). The *in vivo* cell expansion are showed by different cell types such as **(E)** Treg, **(F)** CD8 T and **(G)** NK cells (6 mouse/group).

In the *ex vivo* PBMC expansion assay, as shown in **Figures 5A, B**, IL-2v treatment expanded PBMC to a similar extent as wtIL-2 treatment did, although higher concentrations of IL-2v was required for maximal proliferation. Expansion curves for PBMC treated with 50 nM IL-2v or wtIL-2 demonstrated that the IL-2 variant was similarly effective as the wildtype in promoting the PBMC expansion at 50 nM. Meanwhile, IL-2v expanded both CD8 T cells and NK cells, whereas Treg expansion was significantly down regulated after PBMC *ex vivo* expansion in comparison with wtIL-2 (**Supplementary Table S5**.).

The selective activation profile of the IL-2v was further confirmed *in vivo*. Consistent with the *in vitro* findings, IL-2v at all doses tested did not increase the percentage of CD4+Foxp3+ Tregs, while wtIL-2 resulted in a dose-dependent expansion of Tregs (**Figure 5E**). On the other hand, IL-2v showed dose-dependent activity in promoting the proliferation of CD8 T and NK cell *in vivo* (**Figures 5F, G**) but at a lower level compared to wtIL-2.

During the *ex vivo* cell expansion, the stemness or exhaustion markers of proliferated T cells had been evaluated to determine the level of cell activation. As shown in **Figure 5C**; **Supplementary Table S6**, after 10-day cell expansion, the ratio of CCR7+ CD62L+ CD8 T cells is 41.99% and 27.79% in IL-2v and wtIL-2 groups, respectively. Meanwhile, the population of LAG-3 and TIM-3 double negative CD8 T was significantly up-regulated in IL-2v treated group (**Figure 5D**). These results indicated that IL-2v treated T cells maintained more stemness marker and less exhaustion marker than wtIL-2. To further investigate the mechanism, the genetic transcription of immune regulating pathways had been studied by RNAseq after CD8 T cell or NK cell expansion. In CD8 T cell, as shown in **Supplementary Figures S8A, B**, IL-2v down-regulated Treg marker, FoxP3, and IL-2 associated inflammatory pathway in comparison with wtIL-2, while the transcription of chemokines such as CCL3 and CCL4 were up-regulated. In NK cell, the Treg and exhaustion markers had also been down-regulated by the treatment of IL-2v, while the immune pathway of IL-2 signal and inflammatory response had also been down-regulated similarly as observed in CD8 T cell (data not shown). Moreover, the stemness marker Ki67 had also been monitored during CD8 T and NK cell *in vivo* expansion. As shown in **Supplementary Figure S9**, the high ratio of Ki67^hi^ CD8 T cells/NK cells to Ki67^hi^ Tregs was represented at the high dosage of IL-2v. Therefore, the “βγ-only” IL-2v may have maintained the stemness marker by limiting the activation of exhaustion pathways in CD8 and NK cells.

**Figure 6:**
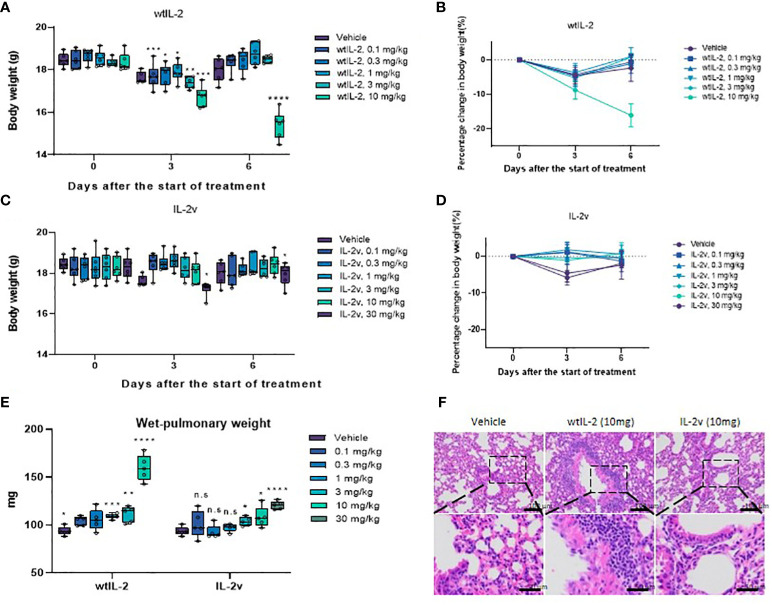
Toxicity study for wtIL-2 and IL-2v. **(A, B)** are the bodyweight measurements after wtIL-2 treatment. **(C, D)** are the bodyweight measurements after IL-2v treatment. **(E)** is the wet-pulmonary weight level after wtIL-2 or IL-2v treatment (6 mouse/group). **(F)** is the IHC result in lung of vehicle group, wtIL-2 group and IL-2v group. The symbols *, **, ***, **** refers to significant differences by the results of p-value of the data, while the ns refers to no significant differences.

We next evaluated *in vivo* tolerability of IL-2v. As shown in **Figures 6A–D**, the mice injected with 10 mg/kg wtIL-2 experienced great weight loss, while those treated with 10 mg/kg or 30 mg/kg IL-2v did not show much significant body weight change. The average wet pulmonary weight from the 30 mg/kg IL-2v-treated group was significantly lower than that from the 10mg/kg wtIL-2-treated group, and comparable to those from the lower dose groups, indicating that the severity of pulmonary edema was much lower in IL-2v treated groups compared with wtIL-2 treated groups (**Figure 6E**). Moreover, serum IL-5, IL-6, IL-10, IFN-γ, TNF-α level after wtIL-2 or IL-2v treatment were monitored as pro-inflammatory markers for toxicity. As shown in **Supplementary Figures S10A-E**, IL-2v treatment did not elevate serum cytokine levels both 6 hours after the first dose and after day 5 in comparison with wtIL-2. The significant reduction of IL-5, IL-6 and IFN-γ, level by IL-2v treatment indicated that less pro-inflammatory immune activation than wtIL-2. The ALT, AST and LDH levels were also monitored to identify the toxicity (**Supplementary Figures S10F-H**). With the high dosage of 10 mg/kg, wtIL-2 represented significant higher level of ALT than IL-2v group. The IHC analysis in the lung and liver corroborated the toxicity of wtIL-2 with evident accumulation of infiltrated immune cells. However, no inflammatory immune cells was observed in the animals treated with the same dose of IL-2v (**Figure 6F**; **Supplementary Figure S10I**). Taken together, these data demonstrate that IL-2v had significantly reduced toxicity than wtIL-2 *in vivo*.

### Bispecific PD-1-IL-2v showed great efficacy and safety in a high-dosed manner without checkpoint inhibitor combination *in vivo*

Because of the unique properties of “βγ-only” IL-2v, such as non-recruitment by any single IL-2 receptors, Treg unfavorable but CD8 T and NK moderate activations, good safety profile *in vivo*, one of the appropriate design is to generate IL-2v a targeting moiety to activated T cell. Therefore, PD-1 is one of the best target so that a bispecific PD-1-IL-2v had been designed and synthesized (**Supplementary Figure S1B**). The IL-2v moiety was fusion expressed on the C-terminal of anti-human PD-1 mAb, both double armed and single armed (knob-into-hole (KIH) bespecific structure (22)) modifications had been expressed and purified while the single armed molecule represented better yield and stability (data not shown). To ensure the function of anti-PD-1 antibody and IL-2v in bispecific molecule, measurement of binding affinity with PD-1 and the *in vitro* activation assays have been generated. As shown in **Supplementary Figure S11**, bispecific PD-1-IL-2v represented similar binding affinity with anti-PD-1 antibody and well functioned of IL-2v in HH cell line.

**Figure 7:**
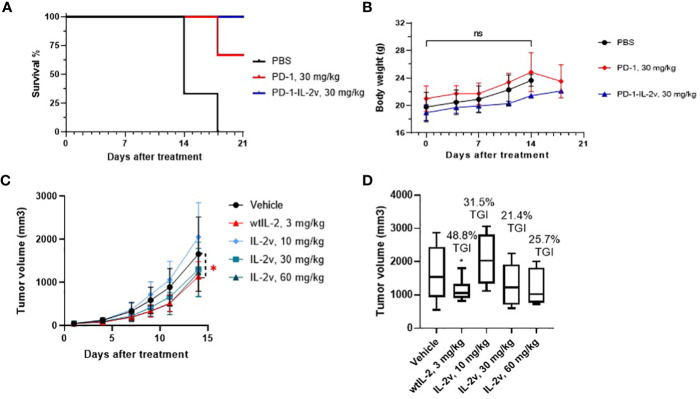
*In vivo* efficacy and safety of PD-1-IL-2v and IL-2v. **(A, B)** showed the *in vivo* efficacy and safety of bispecific PD-1-IL-2v, respectively (3 mouse/group). **(C)** showed the *in vivo* efficacy of IL-2v single agent treatment, which TGI is showed in **(D)** (6 mouse/group). The symbol * refers to significant differences by the results of p-value of the data, while the ns refers to no significant differences.

The anti-tumor efficacy of bispecific PD-1-IL-2v was evaluated with the present of PD-1 antibody as a bench marker in MC38 model. PD-1-IL-2v demonstrated a greater anti-tumor efficacy than PD-1 antibody at the same dose of 30 mg/kg regarding to the survival and body weight data of mice (**Figures 7A, B**). The IHC results showed PD-1-IL-2v group had approximal 3-fold more CD8+ T cell accumulation in solid tumor tissue than PD-1 single dose group, while several CD8+/pSTAT5+ staining in PD-1-IL-2v group which had not existed under PD-1 single agent treatment (**Supplementary Figure S12**). This is also supporting our hypothesis that dual MOAs of PD-1-IL-2v with both PD-1 (CD8 signal) and IL-2 (pSTAT5 signal) T cell activation. To further demonstrate the MOA of PD-1-IL-2v, the IL-2v single agent and the combination treatment of IL-2v and PD-1 antibody had been conducted in the *in vivo* efficacy studies. In the study of IL-2v single agent in the mouse MC38 model, the dosage of 30 mg/kg and 60 mg/kg IL-2v were well tolerated. The result demonstrated tumor growth inhibition (TGI) with 21.4% and 25.7% in the dosage of 30 mg/kg and 60 mg/kg, respectively (**Figures 7C, D**; **Supplementary Figure S13**). IL-2v single agent represented no significant anti-tumor activity compare to wtIL-2 which generated the TGI with 31.5% in 3 mg/kg. In the study of IL-2v and PD-1 antibody combination therapy, all combination groups with the dosage of IL-2v at 3 mg/kg, 10 mg/kg and 30 mg/kg behaved less tumor suppression than single agent PD-1 antibody group, indicating that IL-2v does not help the check-point inhibitor for tumor suppression in the combination treatment (data not shown). Therefore, the molecular fusion of PD-1 antibody and IL-2v to form a bispecific molecule is necessary for “*in-cis*” T cell activation.

## Discussion

**Figure 8:**
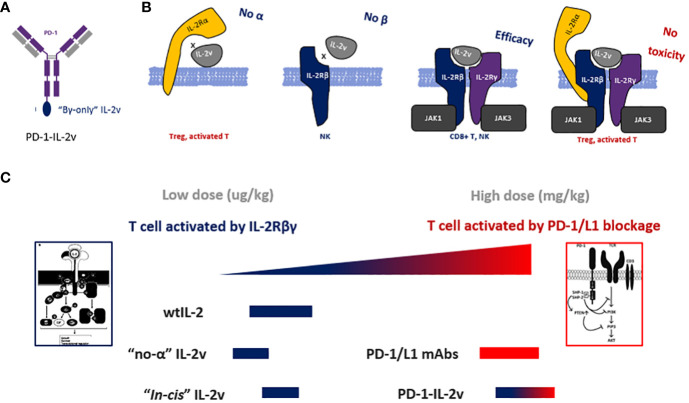
The molecular design and dual-MOAs of bispecific molecule. **(A)** Design and structure of bispecific PD-1-IL-2v. **(B)** The binding pattern of IL-2v with each and the combinations of IL-2 receptors. **(C)** the MOA and dosage of different IL-2 variants and bispecific antibodies.

In this study, “βγ-only” IL-2v was designed to increase the dosage of bispecific PD-1-IL-2v and to possess the dual MOAs (mechanism of action) of PD-1/L1 blockage and “*in-cis*” T cell activation (**Figure 8**). It is technically challenging to remove the IL-2Rβ binding while still maintaining the affinity to IL-2Rβγ complex. In previous studies, mutations of N88R or D20T on IL-2 had been designed to reduce the binding of IL-2Rβ to generate the molecules of BAY 50-4798 or EMD 521873, respectively (8, 23, 24). The reduced binding affinity to IL-2Rβ, however, dramatically reduced the interaction with IL-2Rβγ complex resulting in a poor anti-tumor efficacy. Therefore, to remain the IL-2Rβγ complex interaction and reduce the affinity with single β receptor, our strategy is to generate conformational change in IL-2 by structural shuffling of α-helixes, so that this structural twisted IL-2 variant can disrupt the interaction with IL-2Rβ which has small binding surface, while remain the binding affinity with IL-2Rβγ complex which has relatively large binding surface by rapid switch-back to natural IL-2 conformation. Due to the lack of structural information after structure shuffling, a small library with all linkage (after circular permutation) was used to identify the best candidate. To confirm the structure of IL-2 variant 012, its structure had also been predicted in AlphaFold2 and aligned with wtIL-2. The result showed the perfect alignment of IL-2v 012 against wtIL-2 in IL-2Rβγ binding part. Interestingly, because of the different configuration generated by structural shuffling, the twisted IL-2v has “fast-on/off” binding pattern, which could play an important role in the safety profile as an immune agonist (25) and exhibit very different properties from “no-α” IL-2 variants, which are the most popular IL-2 biased immune agonist variants in recent decade.

“No-α” IL-2 variants have been developed to limit Treg activation, however, several clinical trials revealed that “no-α” IL-2 behaved less tolerance in dose-dependent manner and did not yield better efficacy compared with wtIL-2 (13, 26, 27). Multiple mechanisms may contribute to the failure of “no-α” IL-2 variants. First, the reduction of IL-2Rα binding results in the losing of target function to IL-2Rα+ T cells which in an activated state with the effective with anti-tumor. Second, overstimulation of T cells due to the reduction of Treg-mediated immunosuppression. Third, “no-α” IL-2 variants have “βγ biased” function which efficiently activates NK cells. Other approach, such as “αβ biased” IL-2 variants, has been evaluated in combination with check point inhibitors. “αβ biased” IL-2 variants have increased binding affinity to IL-2Rα to activate Treg with greater degree of immunosuppression. WtIL-2 has been evolved for millions of years to balance the immune system via the selection of IL-2Rα. Biased IL-2 might break this balance by generating either over stimulation or immunosuppression. Thus, to decouple the function of effective T cell and NK activation to avoid of over stimulation or immunosuppression, the unbiased βγ specific binding pattern is required. “βγ-only” IL-2 enables a fine balance of immune system stimulation by reduction of NK activation and Treg suppression via reduces NK overstimulation by eliminating IL-2Rβ binding, and it also abolishs Treg immunosuppression via the absence of IL-2Rα binding. This enables moderate activation of T and NK cells.

In pre-clinical studies and clinical trials, both “βγ biased” and “αβ biased” IL-2 variants represent the normal or lower dosage as a cytokine, because of the recruitment by either IL-2Rβ or IL-2Rα, located on NK or Treg, respectively. Therefore, the bispecific molecule, which *“in-cis”* activates T cell with the moiety of “βγ biased”, “αβ biased” or wtIL-2 isoforms, can only be dosed 10-100x lower concentration than mAb. So they need the combination treatment with checkpoint inhibitors such as PD-1/L1 antibody to achieve a good efficacy. Interestingly, the sequence of PD-1 targeting bispecific molecule is from FDA proved PD-1 antibody whose MOA is blocking PD-1/L1 axis signaling. Thus theoretically, this sequence and its epitope are 1) not the best for “*in-cis*” activating and 2) perturbed by combined PD-1 antibody. Therefore in clinical trial (NCT04303858), the bispecific PD-1-IL-2v RO7284755 was in combination with Atezolizumab (anti-PD-L1) to avoid the competitive of PD-1-epitope binding. The ideal dosage of this bespecific antibody is not a cytokine dosage in ug/kg but a mAb check point inhibitor dosage in mg/kg. T cell “*in-cis*” activating is the second priory to enhance this check point inhibitor and somehow, to potentially generate a secondary immune stimulation on PD-1+ T cells while PD-1 resistance. In this case, PD-1 is a better target than CD8 for “*in-cis*” activation, because targeting PD-1 can not only highlight the group of activated T cell, but also release the immune suppression via PD-1/L1 interaction.

In conclusion, here we report a bispecific antibody PD-1-IL-2v with great safety profile in high dosage and reliable anti-tumor efficacy *in vivo*. The IL-2v moiety is designed as a novel “βγ-only” binding pattern unbiased to each single IL-2R subunit for decoupling their functions to moderately activate T and NK cells. This bispecific antibody possesses the ability to not only block the PD-1/L1 but also moderately stimulate CD8 T cell and NK cell while maintaining their stemness. PD-1-IL-2v has a great potential to be developed as an anti-tumor drug without combination treatment of PD-1/L1 check point inhibitor.

## Data availability statement

The datasets presented in this study can be found in online repositories. The names of the repository/repositories and accession number(s) can be found below: NCBI via accession ID PRJNA1069120.

## Ethics statement

Ethical approval was not required for the studies on humans in accordance with the local legislation and institutional requirements because only commercially available established cell lines were used. The animal study was approved by Institutional animal care and use committee IACUC. The study was conducted in accordance with the local legislation and institutional requirements.

## Author contributions

LZ: Conceptualization, Data curation, Funding acquisition, Investigation, Project administration, Resources, Supervision, Validation, Visualization, Writing – original draft, Writing – review & editing. YJ: Data curation, Investigation, Methodology, Project administration, Supervision, Writing – review & editing. CC: Data curation, Formal analysis, Methodology, Project administration, Software, Validation, Writing – review & editing. YL: Methodology, Project administration, Software, Supervision, Validation, Writing – review & editing. RW: Data curation, Formal analysis, Investigation, Methodology, Validation, Writing – review & editing. CF: Data curation, Formal analysis, Investigation, Methodology, Validation, Writing – review & editing. LC: Data curation, Investigation, Methodology, Validation, Writing – review & editing. SC: Data curation, Formal analysis, Investigation, Methodology, Validation, Writing – review & editing.

## References

[1] Bluestone JA. The yin and yang of interleukin-2-mediated immunotherapy. New Engl J Med. (2011) 365:2129–31. doi: 10.1056/NEJMe1110900 22129258

[2] Boyman O Sprent J. The role of interleukin-2 during homeostasis and activation of the immune system. Nat Rev Immunol. (2012) 12:180–90. doi: 10.1038/nri3156 22343569

[3] Liao W Lin JX Leonard WJ. IL-2 family cytokines: new insights into the complex roles of IL-2 as a broad regulator of T helper cell differentiation. Curr Opin Immunol. (2011) 23:598–604. doi: 10.1016/j.coi.2011.08.003 21889323 PMC3405730

[4] Wang X Rickert M Garcia KC. Structure of the quaternary complex of interleukin-2 with its alpha, beta, and gammac receptors. Science. (2005) 310:1159–63. doi: 10.1126/science.1117893 16293754

[5] Boyman O Kovar M Rubinstein MP Surh CD Sprent J. Selective stimulation of T cell subsets with antibody-cytokine immune complexes. Science. (2006) 311:1924–7. doi: 10.1126/science.1122927 16484453

[6] Krieg C Letourneau S Pantaleo G Boyman O. Improved IL-2 immunotherapy by selective stimulation of IL-2 receptors on lymphocytes and endothelial cells. Proc Natl Acad Sci U S A. (2010) 107:11906–11. doi: 10.1073/pnas.1002569107 PMC2900642 20547866

[7] Huh D Leslie DC Matthews BD Fraser JP Jurek S Hamilton GA. A human disease model of drug toxicity-induced pulmonary edema in a lung-on-a-chip microdevice. Sci Trans Med. (2012) 4:159ra147. doi: 10.1126/scitranslmed.3004249 PMC8265389 23136042

[8] Shanafelt AB Lin Y Shanafelt MC Forte CP Dubois-Stringfellow N Carter C. A T-cell-selective interleukin 2 mutein exhibits potent antitumor activity and is well tolerated in vivo. Nat Biotechnol. (2000) 18:1197–202. doi: 10.1038/81199 11062441

[9] Li Y Strick-Marchand H Lim AI Ren J Masse-Ranson G Dan L. Regulatory T cells control toxicity in a humanized model of IL-2 therapy. Nat Commun. (2017) 8:1762. doi: 10.1038/s41467-017-01570-9 29176694 PMC5701141

[10] Mullard A. Restoring IL-2 to its cancer immunotherapy glory. Nat Rev Drug Discovery. (2021) 20:163–5. doi: 10.1038/d41573-021-00034-6 33603157

[11] Parisi G Saco JD Salazar FB Tsoi J Krystofinski P Puig-Saus C. Persistence of adoptively transferred T cells with a kinetically engineered IL-2 receptor agonist. Nat Commun. (2020) 11:660. doi: 10.1038/s41467-019-12901-3 32005809 PMC6994533

[12] Ptacin JL Caffaro CE Ma L San Jose Gall KM Aerni HR Acuff NV. An engineered IL-2 reprogrammed for anti-tumor therapy using a semi-synthetic organism. Nat Commun. (2021) 12:4785. doi: 10.1038/s41467-021-24987-9 34373459 PMC8352909

[13] Lopes JE Fisher JL Flick HL Wang C Sun L Ernstoff MS. ALKS 4230: a novel engineered IL-2 fusion protein with an improved cellular selectivity profile for cancer immunotherapy. J immunother Cancer. (2020) 8:e000673. doi: 10.1136/jitc-2020-000673 32317293 PMC7204809

[14] Klein C Waldhauer I Nicolini VG Freimoser-Grundschober A Nayak T Vugts DJ. Cergutuzumab amunaleukin (CEA-IL2v), a CEA-targeted IL-2 variant-based immunocytokine for combination cancer immunotherapy: Overcoming limitations of aldesleukin and conventional IL-2-based immunocytokines. Oncoimmunology. (2017) 6:e1277306. doi: 10.1080/2162402X.2016.1277306 28405498 PMC5384349

[15] Ribba B Boetsch C Nayak T Grimm HP Charo J Evers S. Prediction of the optimal dosing regimen using a mathematical model of tumor uptake for immunocytokine-based cancer immunotherapy. Clin Cancer Res. (2018) 24:3325–33. doi: 10.1158/1078-0432.CCR-17-2953 29463551

[16] Levin AM Bates DL Ring AM Krieg C Lin JT Su L. Exploiting a natural conformational switch to engineer an interleukin-2 'superkine'. Nature. (2012) 484:529–33. doi: 10.1038/nature10975 PMC3338870 22446627

[17] Silva DA Yu S Ulge UY Spangler JB Jude KM Labao-Almeida C. *De novo* design of potent and selective mimics of IL-2 and IL-15. Nature. (2019) 565:186–91. doi: 10.1038/s41586-018-0830-7 PMC6521699 30626941

[18] Codarri Deak L Nicolini V Hashimoto M Karagianni M Schwalie PC Lauener L. PD-1-cis IL-2R agonism yields better effectors from stem-like CD8(+) T cells. Nature. (2022) 610:161–72. doi: 10.1038/s41586-022-05192-0 PMC9534752 36171284

[19] Tichet M Wullschleger S Chryplewicz A Fournier N Marcone R Kauzlaric A. Bispecific PD1-IL2v and anti-PD-L1 break tumor immunity resistance by enhancing stem-like tumor-reactive CD8(+) T cells and reprogramming macrophages. Immunity. (2023) 56:162–79.e6. doi: 10.1016/j.immuni.2022.12.006 36630914

[20] Amereh M Akbari M. Immunohistochemistry (IHC) staining of in-vitro cancer cell-generated tumoroids. MethodsX. (2023) 10:102242. doi: 10.1016/j.mex.2023.102242 37346478 PMC10279904

[21] Taube JM Akturk G Angelo M Engle EL Gnjatic S Greenbaum S. The Society for Immunotherapy of Cancer statement on best practices for multiplex immunohistochemistry (IHC) and immunofluorescence (IF) staining and validation. J immunother Cancer. (2020) 8(1):e000155. doi: 10.1136/jitc-2019-000155 32414858 PMC7239569

[22] Ridgway JB Presta LG Carter P. 'Knobs-into-holes' engineering of antibody CH3 domains for heavy chain heterodimerization. Protein Eng. (1996) 9:617–21. doi: 10.1093/protein/9.7.617 8844834

[23] Margolin K Atkins MB Dutcher JP Ernstoff MS Smith JW 2nd Clark JI. Phase I trial of BAY 50-4798, an interleukin-2-specific agonist in advanced melanoma and renal cancer. Clin Cancer Res. (2007) 13:3312–9. doi: 10.1158/1078-0432.CCR-06-1341 17545537

[24] Gillessen S Gnad-Vogt US Gallerani E Beck J Sessa C Omlin A. A phase I dose-escalation study of the immunocytokine EMD 521873 (Selectikine) in patients with advanced solid tumours. Eur J Cancer. (2013) 49:35–44. doi: 10.1016/j.ejca.2012.07.015 22918078

[25] Mao R Kong W He Y. The affinity of antigen-binding domain on the antitumor efficacy of CAR T cells: Moderate is better. Front Immunol. (2022) 13:1032403. doi: 10.3389/fimmu.2022.1032403 36325345 PMC9618871

[26] Bentebibel SE Hurwitz ME Bernatchez C Haymaker C Hudgens CW Kluger HM. and biomarker analysis of NKTR-214, a novel IL2Rbetagamma-biased cytokine, in patients with advanced or metastatic solid tumors. Cancer Discovery. (2019) 9:711–21. doi: 10.1158/2159-8290.CD-18-1495 30988166

[27] Diab A Tannir NM Bentebibel SE Hwu P Papadimitrakopoulou V Haymaker C. Bempegaldesleukin (NKTR-214) plus nivolumab in patients with advanced solid tumors: phase I dose-escalation study of safety, efficacy, and immune activation (PIVOT-02). Cancer Discovery. (2020) 10:1158–73. doi: 10.1158/2159-8290.CD-19-1510 32439653

