# Supplementary material for: A novel dual mechanism-of-action bispecific PD-1-IL-2v armed by a “βγ-only” interleukin-2 variant
Source: Front Immunol. 2024 Apr 4;15:1369376. doi: 10.3389/fimmu.2024.1369376 (PMC11024467; doi:10.3389/fimmu.2024.1369376)
Supplement: Supplementary file 1 [file DataSheet_1.pdf]

Supplementary Materials

A novel dual mechanism-of-action bispecific PD-1-IL-2v armed by a “βγ-only” interleukin-2 variant

Yongji Jiang[1†], Chuyuan Chen[1†], Yuan Liu[1†], Rong Wang[1], Chuan Feng[1], Lili Cai[1], Shuang Chang[1], Lei Zhao[1]*

[1]Cure Genetics Co., LTD, Suzhou, Jiangsu, P.R.China

*Corresponding author. Email: lei.zhao@curegenetics.com

† These authors contributed equally to this work

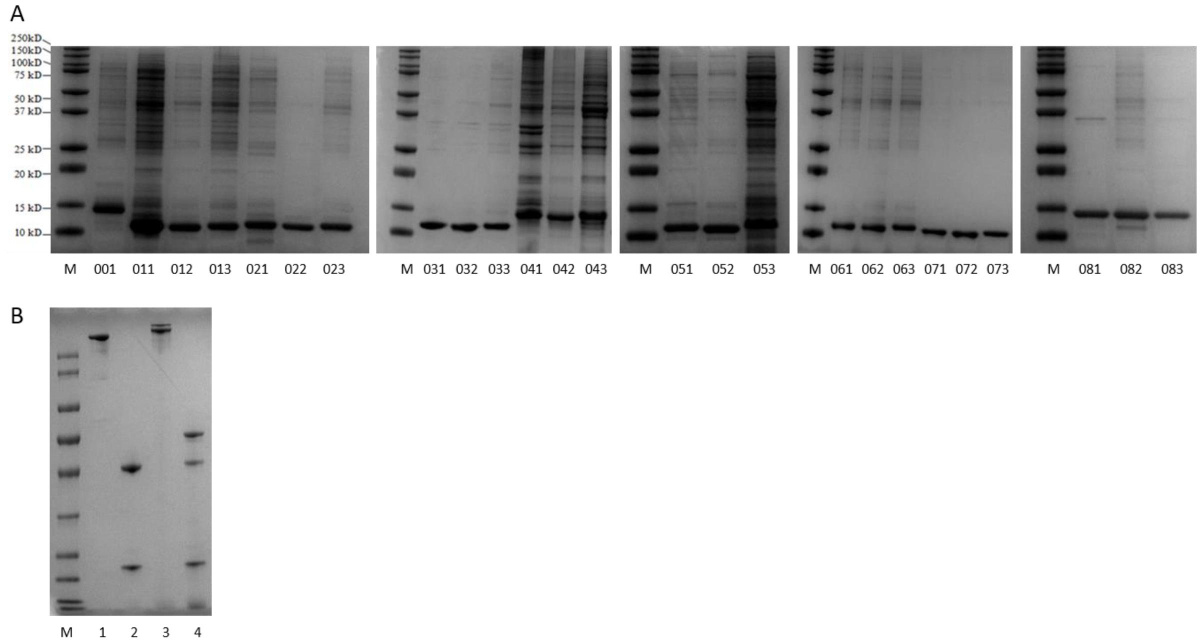

Fig. S1.

(A) Expression and purification of IL-2 variants and wtIL-2. The number of each lane represents the molecule of IL-2v or wtIL-2. **(B)** Expression and purification of PD-1 mAb and bispecific PD-1-IL-2v. Lane 1 and 2 are the natural form and denatured form of PD-1 mAb, respectively. Lane 3 and 4 are the natural form and denatured form of bispecific PD-1-IL-2v, respectively.

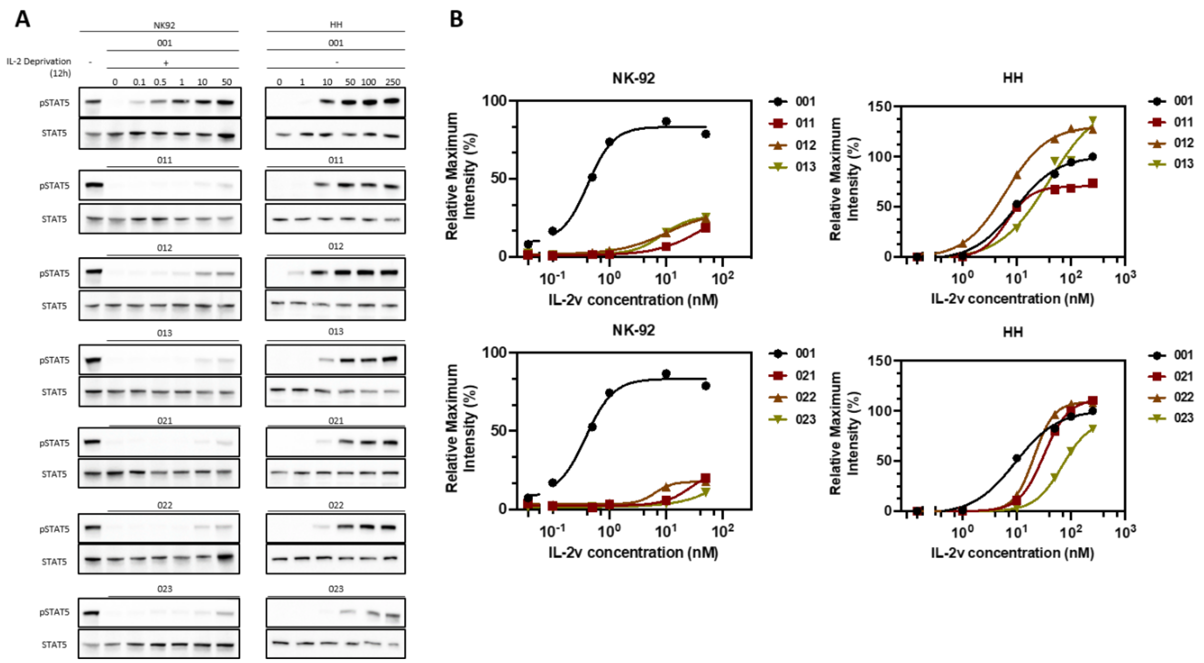

Fig. S2.

In vitro validation of STAT5 activation in NK-92 and HH, 011, 012, 013, 021, 022 and 023. **(A)** The figure from WB; **(B)** the fitting curve from WB results.

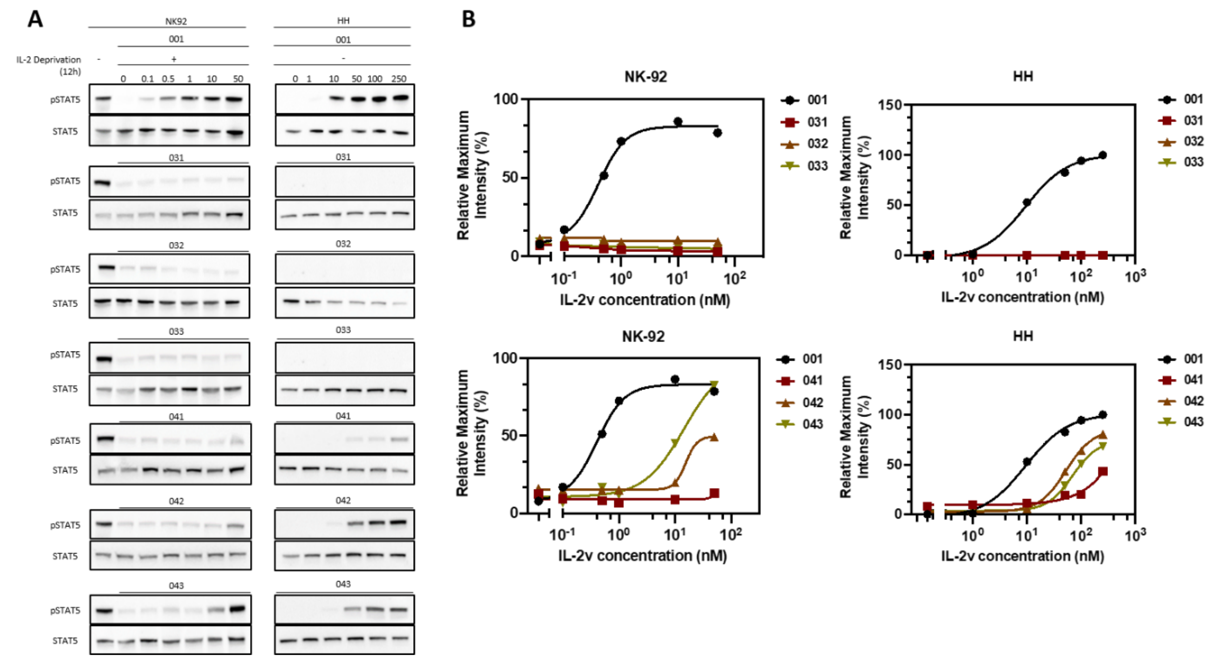

Fig. S3.

In vitro validation of STAT5 activation in NK-92 and HH, 031, 032, 033, 041, 042 and 043. **(A)** The figure from WB; **(B)** the fitting curve from WB results.

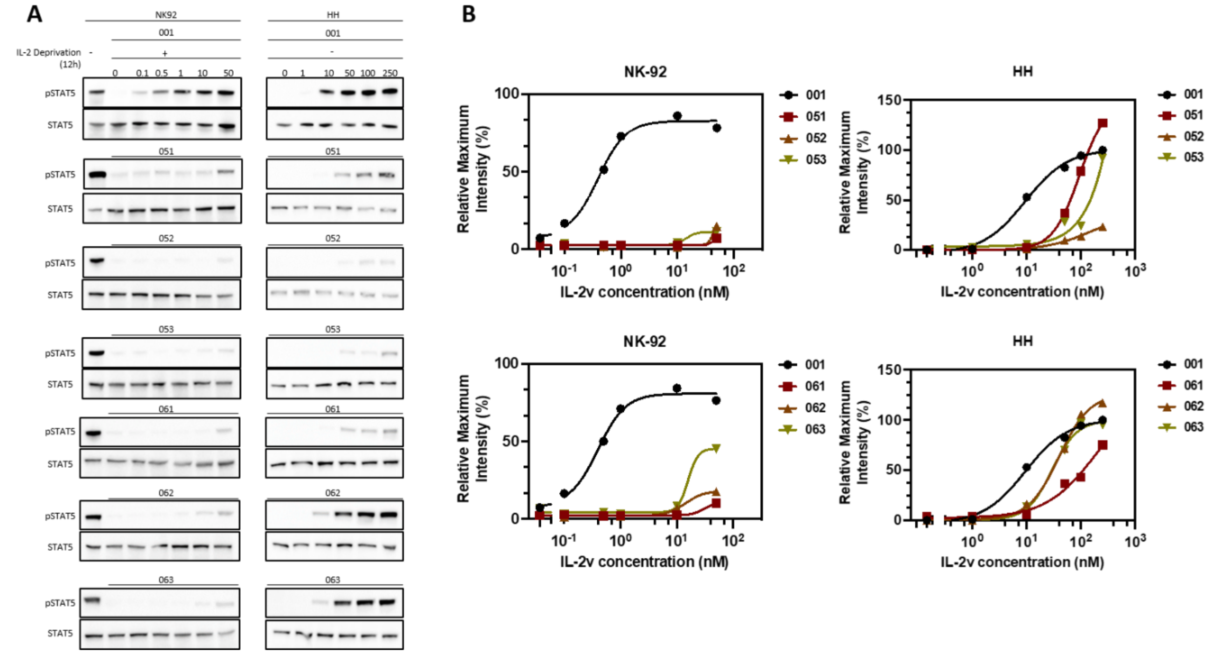

Fig. S4.

In vitro validation of STAT5 activation in NK-92 and HH, 051, 052, 053, 061, 062 and 063. **(A)** The figure from WB; **(B)** the fitting curve from WB results.

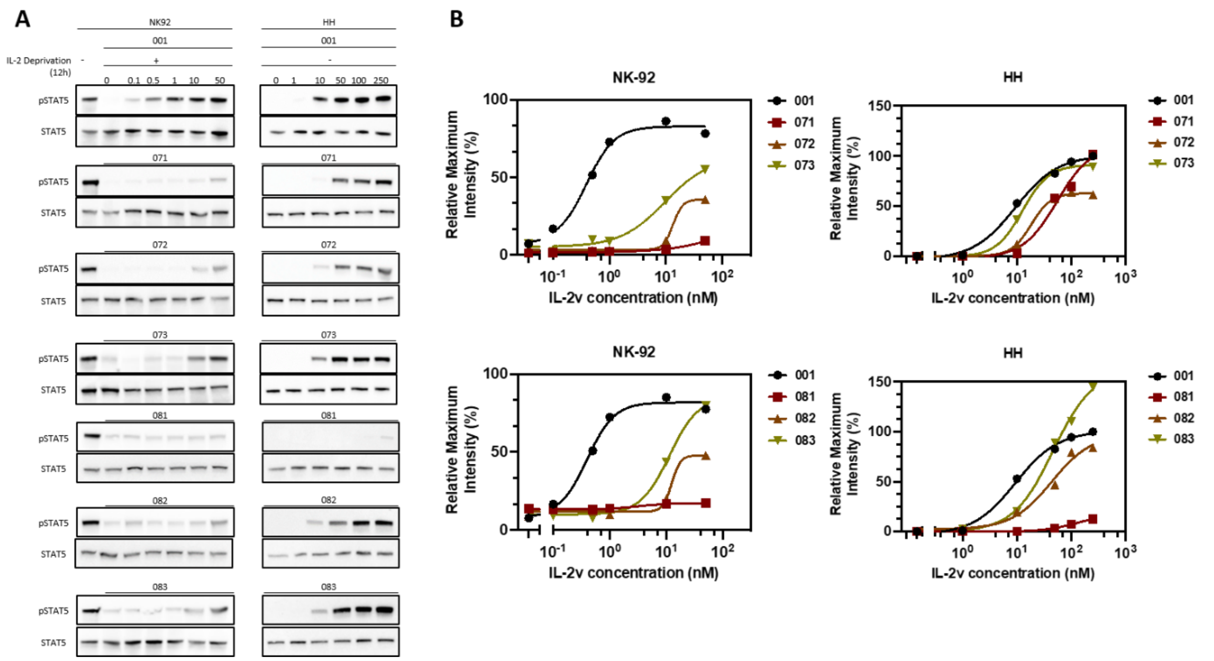

Fig. S5.

In vitro validation of STAT5 activation in NK-92 and HH, 071, 072, 073, 081, 082 and 083. **(A)** The figure from WB; **(B)** the fitting curve from WB results.

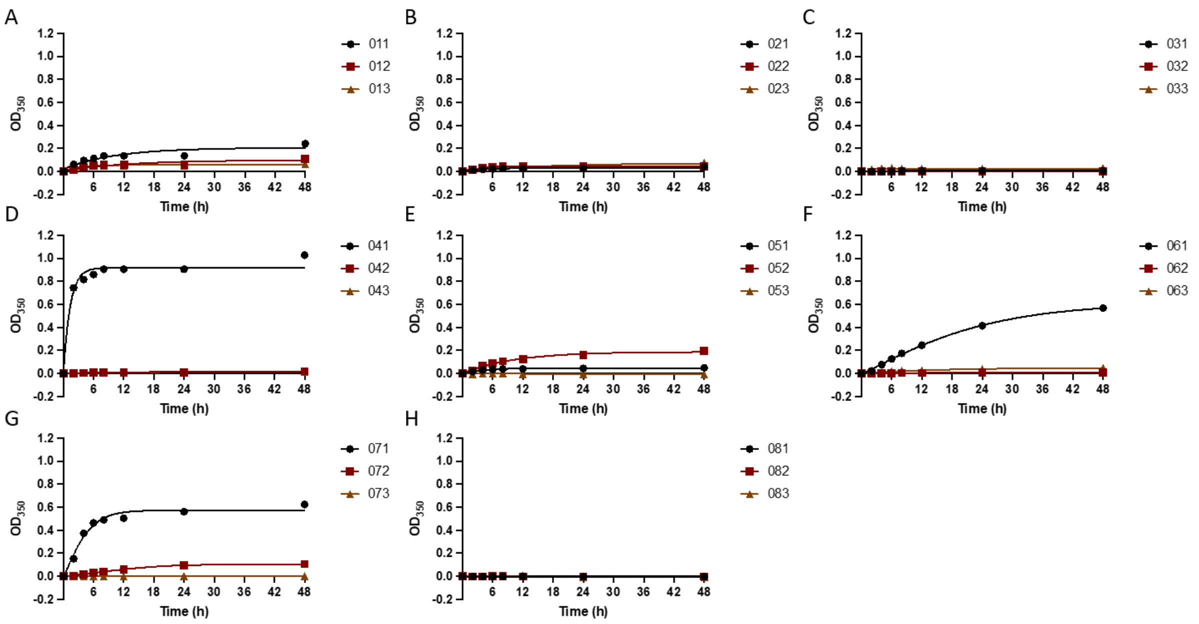

Fig. S6.

Thermo stability of IL-2 variants. **(A)** to **(H)** represent all IL-2 variants in groups.

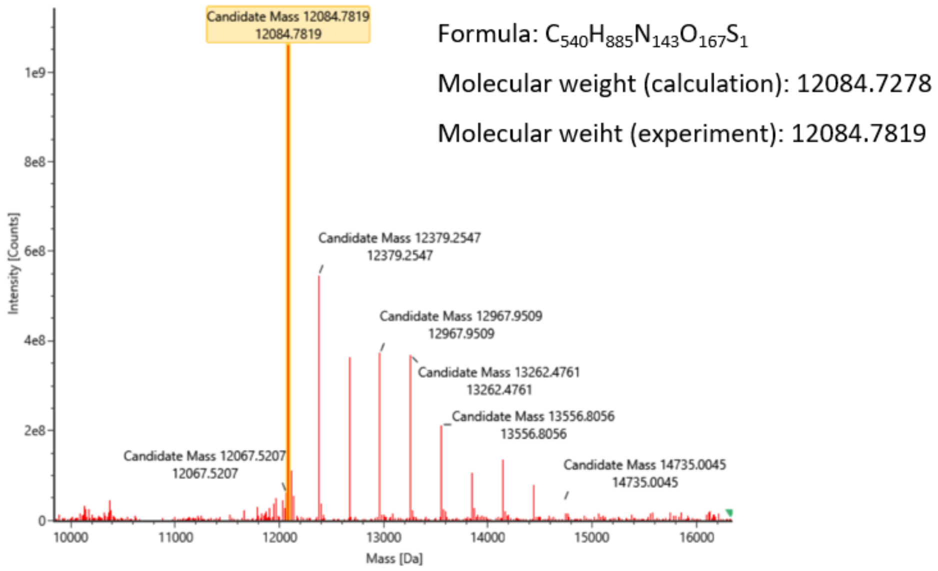

Fig. S7.

Q-Tof result for 012 variant.

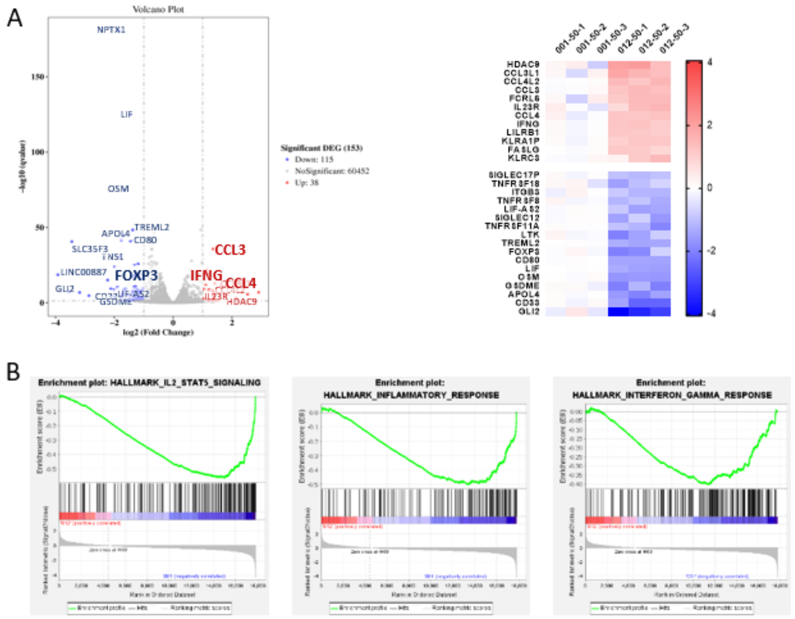

NES: -2.01
P value: 0.000
FDR Q value: 0.001

NES: -1.77
P value: 0.000
FDR Q value: 0.003

NES: -1.44
P value: 0.001
FDR Q value: 0.033

Fig. S8.

RNAseq results of CD8 T cells in **(A)** volcano map and heat map of gene up/down regulation and **(B)** up/down regulation of specific gene pathways (3 replicates/group).

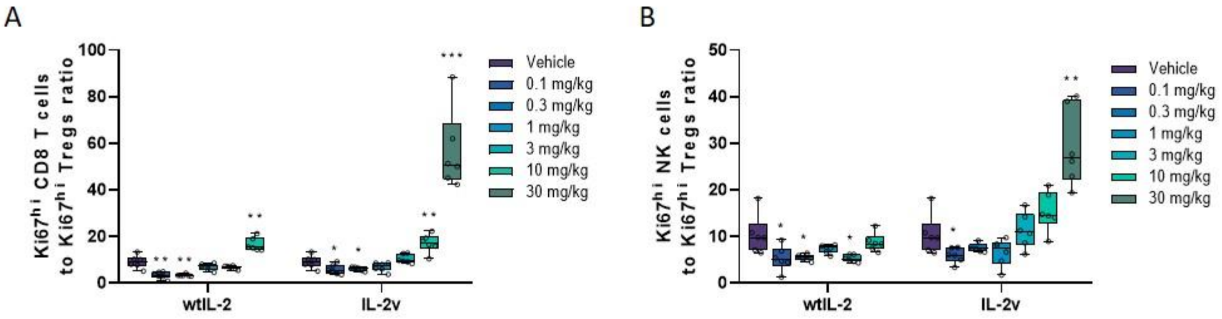

Fig. S9.

(**A**) The ratio of Ki67^{hi} CD8 T cells to Ki67^{hi} Tregs and (**B**) the ratio of Ki67^{hi} NK cells to Ki67^{hi} Tregs after wtIL-2 or IL-2v treated in vivo cell expansion (6 mouse/group).

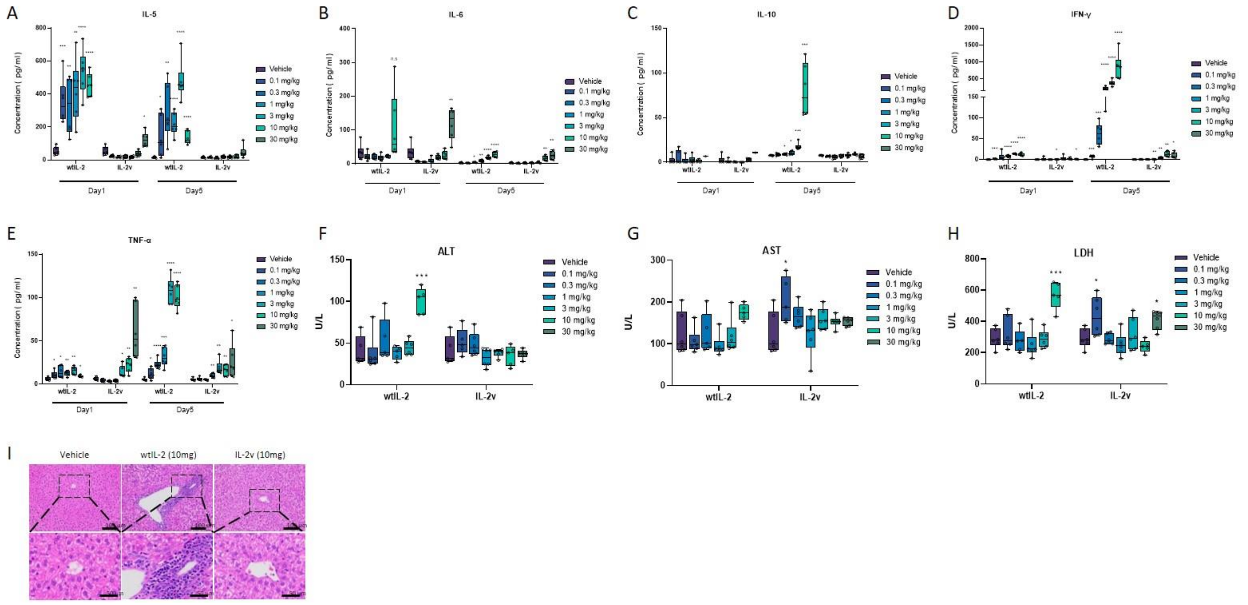

Fig. S10.

The serum levels of (**A**) IL-5, (**B**) IL-6, (**C**) IL-10, (**D**) IFN-γ, (**E**) TNF-α, (**F**) ALT, (**G**) AST, (**H**) LDH (6 mouse/group). (**I**) is the IHC result in liver.

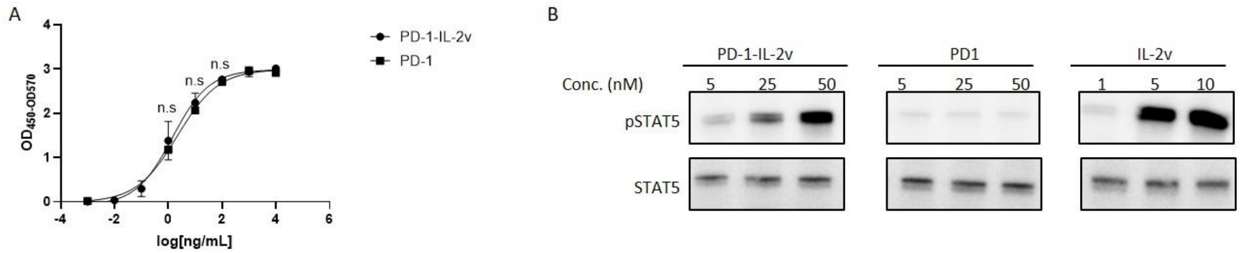

Fig. S11.

In vitro validation of PD-1-IL-2v. **(A)** Binding affinity with human PD-1 monitored by ELISA (3 replicates/group). **(B)** WB result for IL-2v in vitro activity in HH cell line.

A

Name	PD-1-IL-2v	PD-1
Total Cells	7143	4029
pStat5 Positive Cells	102	70
CD8 Positive Cells	220	71
CD8+pStat5 Cells	7	0
DAPI Avg Cell Intensity	32.389828	28.005377
pStat5 Avg Cell Intensity	14.183987	14.842975
CD8 Avg Cell Intensity	5.55934	5.646183

B

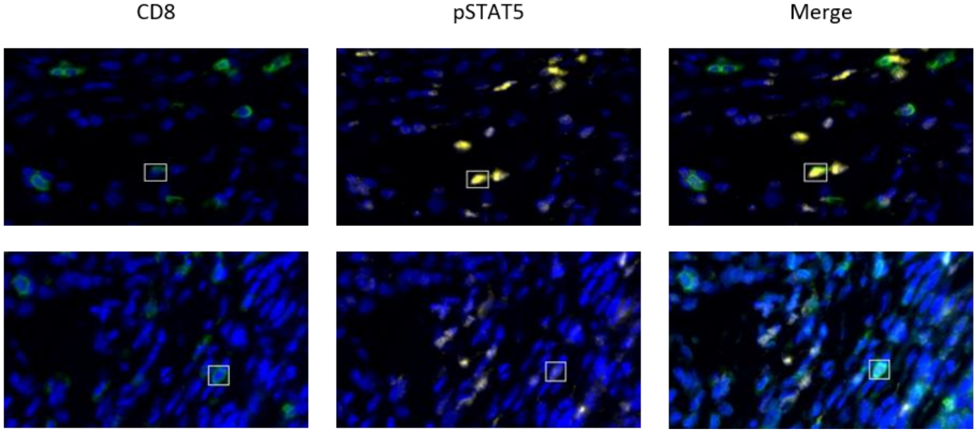

Fig. S12.

(A) Statistics data of IHC sample from the solid tumor in PD-1-IL-2v group and in PD-1 group.

(B) The multi-color staining of CD8 and pSTAT5.

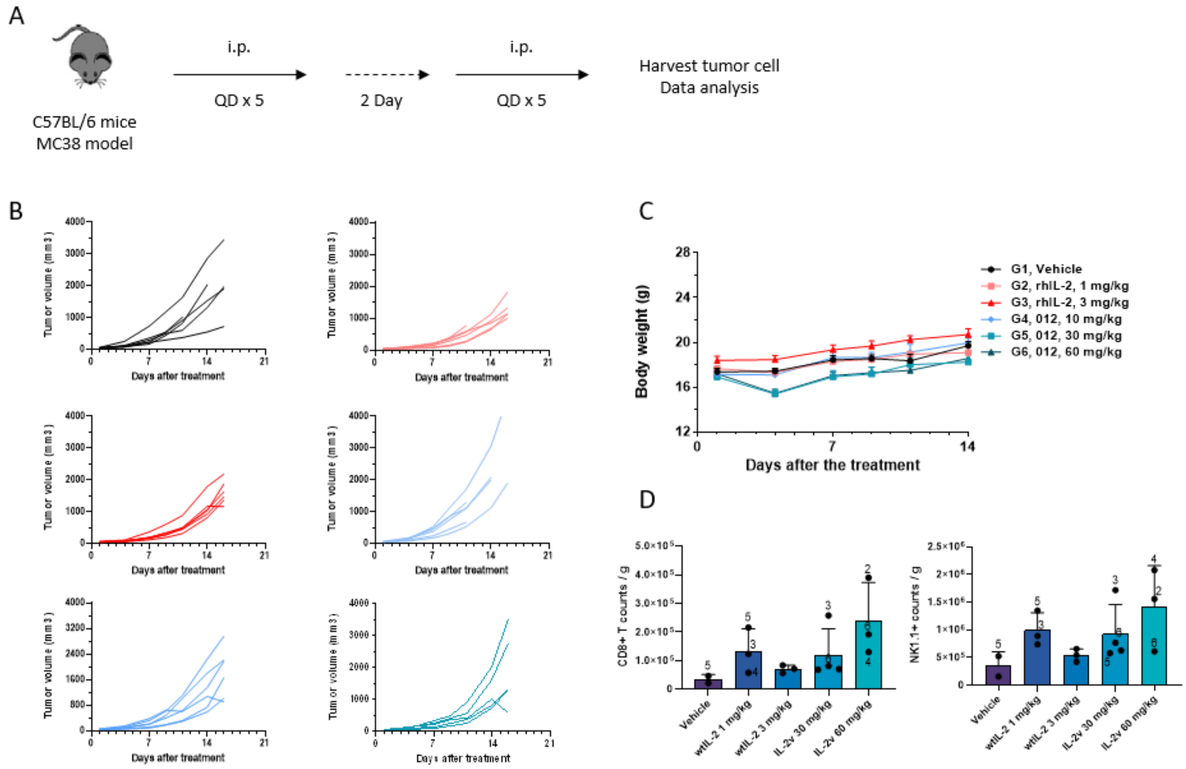

Fig. S13.

(A) the design of IL-2v single agent in vivo efficacy study. **(B)** showed the tumor progression in each single mouse. The body weight of each group of mice is showed in **(C)**. The CD8 T and NK infiltration in solid tumor are showed in **(D)**.

Table S1.

The amino-acid sequence of wtIL-2 and IL-2 variants Type or paste caption here.

IL-2 and variants	Amino Acid Sequences
001 (wild-type IL-2)	APTSSSTKKTQLQLEHLLLDLQMILNGINNYKNPKLTRML TFKFYMPKKATELKHLQCLEEELKPLEEVLNLAQSKNFH LRPRDLISNINVIVLELKGSETTFMCEYADETATIVEFLNR WITFCQSIISTLT
011	PTSSSTKKTQLQLEHLLLDLQMILNGINNYKNPLRPRDLIS NINVIVLELKGSETTELKHLQSLEEELKPLEEVLNLAQSK NFHTIVEFLNRWITFSQSIISTLT
012	PTSSSTKKTQLQLEHLLLDLQMILNGINNYKNPLRPRDLIS NINVIVLELKGSETTELKHLQSLEEELKDLEEVLNLAQSK NFHTIVEFLNRWITFSQSIISTLT
013	PTSSSTKKTQLQLEHLLLDLQMILNGINNYKNPLRPRDLIS NINVIVLELKGSETTELKHLQSLEEELKDLEEVLNLAQSK NFHTIVEKLNRWITKSQSIISTLT
021	LRPRDLISNINVIVLELKGSETTELKHLQSLEEELKPLEEVL NLAQSKNFHTIVEFLNRWITFSQSIISTLTGGGGSGGGGSP TSSSTKKTQLQLEHLLLDLQMILNGIN
022	LRPRDLISNINVIVLELKGSETTELKHLQSLEEELKDLEEV LNLAQSKNFHTIVEFLNRWITFSQSIISTLTGGGGSGGGGS PTSSSTKKTQLQLEHLLLDLQMILNGIN
023	LRPRDLISNINVIVLELKGSETTELKHLQSLEEELKDLEEV LNLAQSKNFHTIVEKLNRWITKSQSIISTLTGGGGSGGGG SPTSSSTKKTQLQLEHLLLDLQMILNGIN

031	ELKHLQSLEEELKPLEEVLNLAQSKNFHTIVEFLNRWITFSQSIISTLTGGGGSGGGGSPTSSSTKKTQLQLEHLLLDLQMILNGINNYKNPLRPRDLISNINVIVLELKG
032	ELKHLQSLEEELKDLEEVLNLAQSKNFHTIVEFLNRWITFSQSIISTLTGGGGSGGGGSPTSSSTKKTQLQLEHLLLDLQMILNGINNYKNPLRPRDLISNINVIVLELKG
033	ELKHLQSLEEELKDLEEVLNLAQSKNFHTIVEKLNRWITKSQSIISTLTGGGGSGGGGSPTSSSTKKTQLQLEHLLLDLQMILNGINNYKNPLRPRDLISNINVIVLELKG
041	TIVEFLNRWITFSQSIISTLTGGGGSGGGGSPTSSSTKKTQLQLEHLLLDLQMILNGINNYKNPLRPRDLISNINVIVLELKGSETTELKHLQSLEEELKPLEEVLN
042	TIVEFLNRWITFSQSIISTLTGGGGSGGGGSPTSSSTKKTQLQLEHLLLDLQMILNGINNYKNPLRPRDLISNINVIVLELKGSETTELKHLQSLEEELKDLEEVLN
043	TIVEKLNRWITKSQSIISTLTGGGGSGGGGSPTSSSTKKTQLQLEHLLLDLQMILNGINNYKNPLRPRDLISNINVIVLELKGSETTELKHLQSLEEELKDLEEVLN
051	PTSSSTKKTQLQLEHLLLDLQMILNGINNYKNPTIVEFLNRWITFSQSIISTLTGGGGSGGGGSELKHLQSLEEELKPLEEVLNLAQSKNFHLRPRDLISNINVIVLELKG
052	PTSSSTKKTQLQLEHLLLDLQMILNGINNYKNPTIVEFLNRWITFSQSIISTLTGGGGSGGGGSELKHLQSLEEELKDLEEVLNLAQSKNFHLRPRDLISNINVIVLELKG
053	PTSSSTKKTQLQLEHLLLDLQMILNGINNYKNPTIVEKLNRWITKSQSIISTLTGGGGSGGGGSELKHLQSLEEELKDLEEVLNLAQSKNFHLRPRDLISNINVIVLELKG

061	TIVEFLNRWITFSQSIISTLTGGGGSGGGGSELKHLQSLEE ELKPLEEVLNLAQSKNFHLRPRDLISNINVIVLELKGSETT PTSSSTKKTQLQLEHLLLDLQMILNGIN
062	TIVEFLNRWITFSQSIISTLTGGGGSGGGGSELKHLQSLEE ELKDLEEVLNLAQSKNFHLRPRDLISNINVIVLELKGSETT PTSSSTKKTQLQLEHLLLDLQMILNGIN
063	TIVEKLNRWITKSQSIISTLTGGGGSGGGGSELKHLQSLEE ELKDLEEVLNLAQSKNFHLRPRDLISNINVIVLELKGSETT PTSSSTKKTQLQLEHLLLDLQMILNGIN
071	ELKHLQSLEEELKPLEEVLNLAQSKNFHLRPRDLISNINVI VLELKGSETTPTSSSTKKTQLQLEHLLLDLQMILNGINNY KNPTIVEFLNRWITFSQSIISTLT
072	ELKHLQSLEEELKDLEEVLNLAQSKNFHLRPRDLISNINVI VLELKGSETTPTSSSTKKTQLQLEHLLLDLQMILNGINNY KNPTIVEFLNRWITFSQSIISTLT
073	ELKHLQSLEEELKDLEEVLNLAQSKNFHLRPRDLISNINVI VLELKGSETTPTSSSTKKTQLQLEHLLLDLQMILNGINNY KNPTIVEKLNRWITKSQSIISTLT
081	LRPRDLISNINVIVLELKGSETTPTSSSTKKTQLQLEHLLLD LQMILNGINNYKNPTIVEFLNRWITFSQSIISTLTGGGGSG GGGSELKHLQSLEEELKPLEEVLN
082	LRPRDLISNINVIVLELKGSETTPTSSSTKKTQLQLEHLLLD LQMILNGINNYKNPTIVEFLNRWITFSQSIISTLTGGGGSG GGGSELKHLQSLEEELKDLEEVLN
083	LRPRDLISNINVIVLELKGSETTPTSSSTKKTQLQLEHLLLD LQMILNGINNYKNPTIVEKLNRWITKSQSIISTLTGGGGSG GGGSELKHLQSLEEELKDLEEVLN

Table S2.

Expression, stability, and bioactivity of IL-2 variants.

IL-2 variants	Expression (mg/L)	Stability (OD350)	Bioactivity
011	79.22	++	+++
012	86.91	+++	+++
013[a]	82.98	+++	+++
021	94.88	++	+++
022	96.71	++	+++
023	61.43	+	++
031	120.62	+	-
032	164.26	+++	-
033	109.57	+++	-
041	40.99	+	+
042	44.38	+++	++
043	47.97	+++	++
051	88.72	+++	++
052	101.42	+	+
053[a]	76.04	+++	+
061	67.11	+	+
062	110.21	+++	+++
063	54.69	+++	++
071	168.96	+	++
072	140.18	++	+++

073[a]	246.55	+++	+++
081	Very Low	+++	+
082	59.63	+++	+++
083	76.52	+++	+++

a: precipitation during concentration

Table S3.

Binding affinity and kinetics of wild-type IL-2 and IL-2 variant 012 to IL-2Rα, IL-2Rβ, common γ receptor, IL-2Rβγ, and IL-2Rαβγ measured by SPR.

Ligand	Capture level (RU)	Analyte	Analyte Conc.	Ka (1/ms)	Kd (1/s)	KD (M)	Rmax (RU)	Fit model
IL-2Rα	270.3	001	0.977-125 nM	NA	NA	4.75E-08	36.5	Steady state affinity
IL-2Rα	301.3	012	0.977-250 nM	No binding				
IL-2Rβ	317.4	001	7.813-250 nM	2.09E +05	1.86 E-02	8.91E-08	97.8	1:1 binding
IL-2Rβ	289.2	012	7.813-250 nM	No binding				
IL-2Rγ	36/9.4	001	0.977-500 nM	No binding				
IL-2Rγ	301.5	012	0.977-500 nM	No binding				
IL-2Rβγ	523.2	001	0.488-62.5 nM	6.70E +05	3.11 E-04	4.63E-10	57.0	1:1 binding
IL-2Rβγ	542.8	012	0.244-15.625 nM	2.28E +06	1.06 E-03	4.67E-10	29.8	1:1 binding
IL-2Rαβγ	625.6	001	0.977-31.25 nM	9.11E +06	3.86 E-04	4.23E-11	63.0	1:1 binding

IL-2Rαβγ	685.2	012	0.977-31.25 nM	1.27E+06	7.32E-04	5.75E-10	36.3	1:1 binding

Table S4.

Potency of STAT5 activation by IL-2v and wtIL-2 in selected human and mouse lymphocyte populations.

Lymphocyte population	Human PBMC			Mouse Splenocytes		
	wtIL-2 EC_{50} (nM)	IL-2v EC_{50} (nM)	Ratio	wtIL-2 EC_{50} (nM)	IL-2v EC_{50} (nM)	Ratio
Tregs	0.1122	7.909	70.5	0.3189	52.02	163.1
CD8 T cells	5.942	18.59	3.1	44.35	91.77	2.1
NK cells	3.098	5.11	1.6	28.57	84.02	2.9

Table S5.

The cell percentage and number after ex vivo cell expansion by 50 nM wtIL-2 or 50 nM IL-2v.

		wtIL-2 (50 nM)			IL-2v (50 nM)			t test	
Sample (No.)		1	2	3	1	2	3	p-value	p-value summary
Percentage (%) (Number)	T cell	97.71 (9.56E7)	98.66 (8.85E7)	98.23 (9.28E7)	98.30 (9.66E7)	98.45 (1.01E8)	98.00 (1.02E8)	0.8777	n.s.
	Treg	1.63 (1.60E6)	2.04 (1.83E6)	1.58 (1.49E6)	1.16 (1.14E6)	1.02 (1.04E6)	0.99 (1.03E6)	0.0110	*
	CD4 T	32.22 (3.15E7)	31.64 (2.84E7)	32.16 (3.03E7)	30.87 (3.03E7)	30.31 (3.12E7)	29.49 (3.06E7)	0.0156	*
	CD8 T	55.62 (5.44E7)	57.85 (5.19E7)	56.62 (5.34E7)	57.28 (5.63E7)	58.91 (6.06E7)	58.25 (6.05E7)	0.1441	n.s.
	NK	0.22 (2.16E5)	0.09 (8.2E4)	0.11 (9.95E4)	0.13 (1.29E5)	0.13 (1.29E5)	0.24 (2.48E5)	0.6507	n.s.

Table S6.

The percentage of stemness and exhaustion markers after ex vivo cell expansion by 50 nM wtIL-2 or 50 nM IL-2v.

		wtIL-2 (50 nM)			IL-2v (50 nM)		
Sample (No.)		1	2	3	1	2	3
Percentage (%)	CD62L^{+}/CCR7^{+}	27.38	28.34	27.66	42.42	42.13	41.43
	CD62L^{-}/CCR7^{-}	9.66	9.07	8.83	13.12	14.10	15.52
	TIM-3^{-}/LAG-3^{-}	32.53	32.11	43.68	50.02	48.79	46.80
	TIM-3^{+}/LAG-3^{+}	9.81	9.62	7.23	6.49	6.15	6.49
